# Supplementary material for: Thermal migration towards constructing W-W dual-sites for boosted alkaline hydrogen evolution reaction
Source: Nat Commun. 2022 Feb 9;13:763. doi: 10.1038/s41467-022-28413-6 (PMC8828749; doi:10.1038/s41467-022-28413-6)
Supplement: Supplementary file 1 — Supplementary Information [file 41467_2022_28413_MOESM1_ESM.pdf]

**Supplementary Information**

# Thermal migration towards constructing W-W dual-sites for boosted alkaline hydrogen evolution reaction

Zhigang Chen[1†], Yafeng Xu[2†], Ding Ding[1], Ge Song[1], Xingxing Gan[1], Hao Li[1], Wei Wei[1], Jian Chen[1], Zhiyun Li[1], Zhongmiao Gong[1], Xiaoming Dong[1], Chengfeng Zhu[1], Nana Yang[1], Jingyuan Ma[3], Rui Gao[4], Dan Luo[4], Shan Cong[1], Lu Wang[2], Zhigang Zhao[1]*, Yi Cui[1]*

[1]Vacuum Interconnected Nanotech Workstation, Suzhou Institute of Nano-Tech and Nano-Bionics, Chinese Academy of Sciences, Suzhou 215123, China.

[2]Institute of Functional Nano & Soft Materials (FUNSOM), Jiangsu Key Laboratory for Carbon-Based Functional Materials & Devices, Soochow University, Suzhou, Jiangsu 215123, China.

[3]Shanghai Synchrotron Radiation Facility (SSRF), Shanghai Advanced Research Institute, Chinese Academy of Sciences, 201204, China.

[4]Department of Chemical Engineering, Waterloo Institute for Nanotechnology, Waterloo Institute for Sustainable Energy, University of Waterloo, Waterloo, Ontario N2L 3G1, Canada.

[†]These authors contributed equally to this work.

*Corresponding authors. Email: zgzhao2011@sinano.ac.cn; ycui2015@sinano.ac.cn

## Supplementary Figures

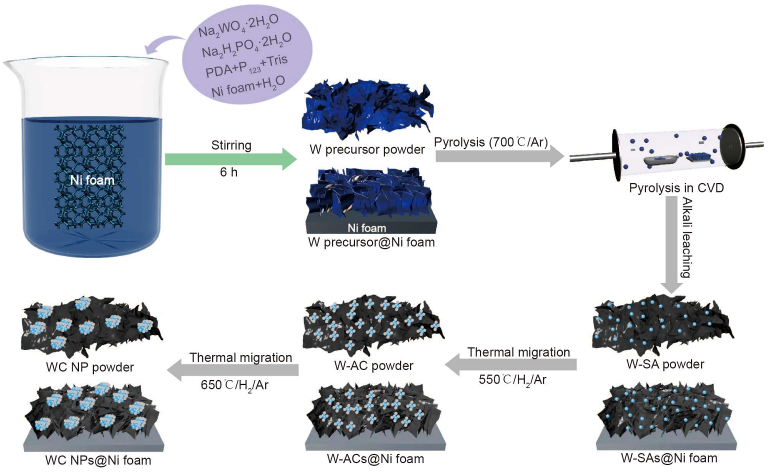


**Supplementary Figure 1**. The synthesis of various tungsten species at different temperature. Schematic illustration of the preparation of tungsten-based powders and Ni foam coated with carbon-supported tungsten species. Tungsten single atoms (W-SAs) are obtained by pyrolyzing the W precursors at 700 °C for 2 h in an Ar atmosphere (1 bar), followed by alkaline leaching in 6 M KOH solution for 3 days. Tungsten atomic clusters (W-ACs) and tungsten carbide nanoparticles (WC NPs) are synthesized by treating the W-SAs at high temperatures of 550 and 650 °C for 30 min in an $Ar/H_2$ atmosphere, respectively.

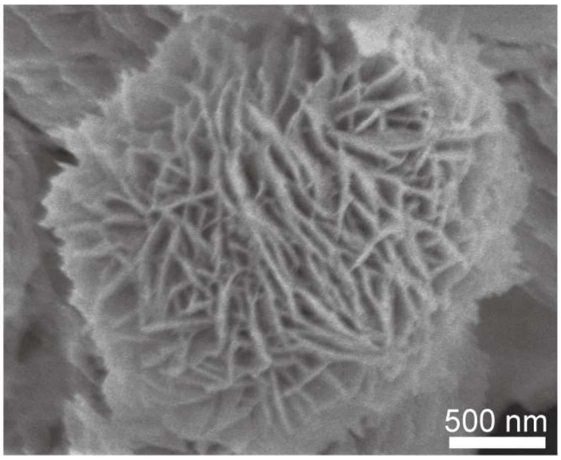


**Supplementary Figure 2**. The morphological observation of W precursors. Typical scanning electron microscopy (SEM) image of W precursors. The as-obtained W precursors exhibit flower-like morphology with vertically grown two-dimensional nanosheets.

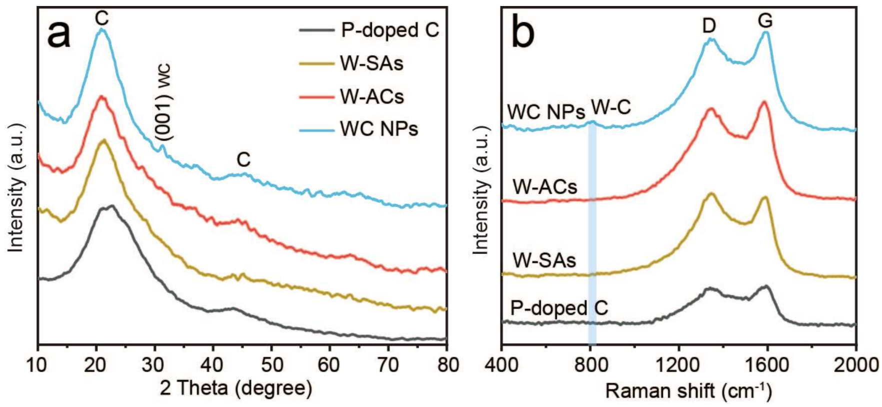


**Supplementary Figure 3**. Structural characterization of the as-obtained tungsten species. (a) X-ray diffraction (XRD) patterns and (b) Raman spectra of P-doped C, W-SAs, W-ACs, and WC NPs, respectively.

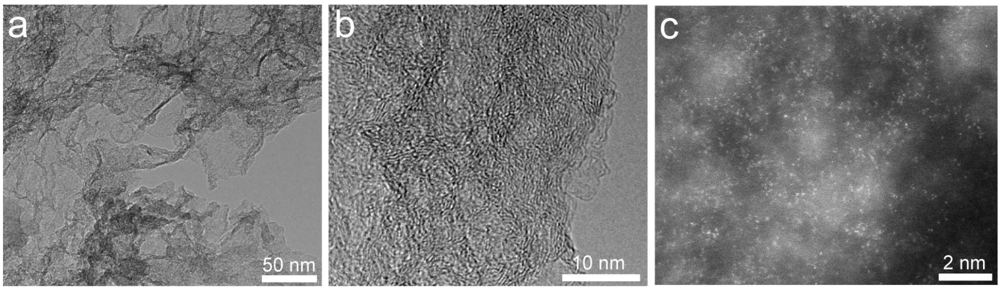


**Supplementary Figure 4**. Morphological characterization of the as-obtained tungsten species. (a) Low- and (b) high-magnification transmission electron microscopy (TEM) images of W-SAs. (c) Atomic-level high-angle annular dark-field scanning transmission electron microscopy (HAADF-STEM) image of W-SAs.

Supplementary Fig. 4a exhibits a nanosheet morphology of the carbon substrates. Only carbon layers without any nanoparticles can be seen in the HRTEM (scale bar: 10 nm) image of W-SAs, because it is challenging to observe ultrasmall nanocrystals (i.e., diameters less than 1 nm) using TEM technique (Supplementary Fig. 4b). However, HAADF-STEM image of W-SAs confirms the existence of the tungsten single atoms with bright spots corresponding to the heavy W atoms (Supplementary Fig. 4c).

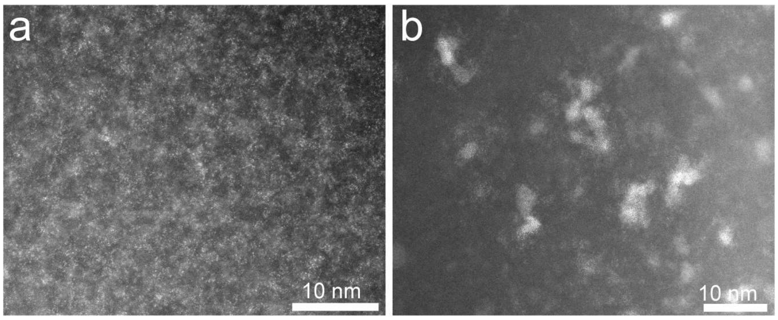


**Supplementary Figure 5**. Morphological observation of W-ACs and WC NPs. The HAADF-STEM images of (a) W-ACs and (b) WC NPs.

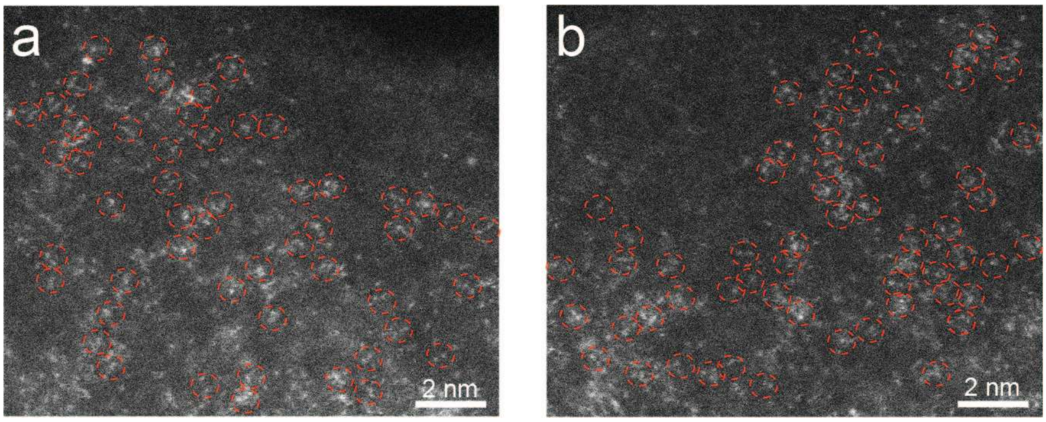


**Supplementary Figure 6**. Morphological observation of W-W dual-atoms. HAADF-STEM images of (a, b) W-ACs. The HAADF-STEM images clearly evidences that a large proportion of the bright spots are adjacent to each other and presented a W-W dimer structures, as indicated by the red dashed circles

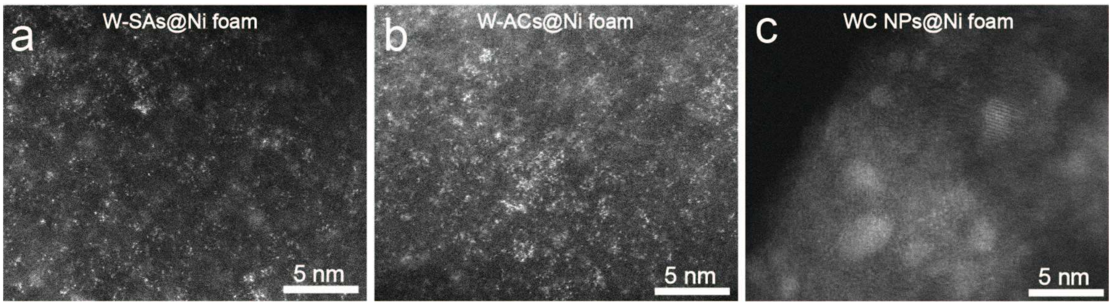


**Supplementary Figure 7**. Morphological observation of the as-obtained three types of tungsten species. The HAADF-STEM images of (a) W-SAs, (b) W-ACs, and (c) WC NPs extracted from Ni foam by sonication. HAADF-STEM images confirms that the tungsten species extracted from Ni foam by sonication are the same to those of the powder samples.

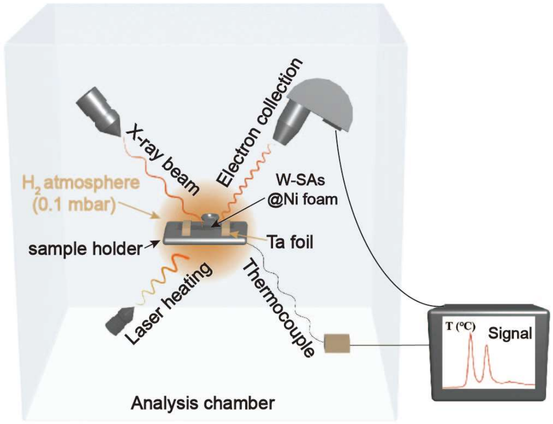


**Supplementary Figure 8**. Near ambient pressure X-ray photoelectron spectroscopy (NAP-XPS) setup. Schematic illustration of the in situ variable-temperature NAP-XPS measurements.

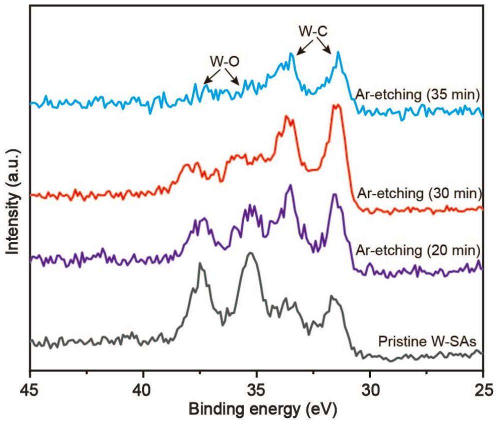


**Supplementary Figure 9**. The surface cleaning of W-SA parent material by Ar-etching method. W *4f* core-level X-ray photoelectron (XPS) spectra of W-SAs pre-treated by Ar-etching method for different periods. The W 4f XPS spectra of W-SAs exhibit a sharp decrease of W-O signals when increasing the Ar-etching time. However, the longer treating times (35 min) affects the intrinsic W-C carbide structure. Thus, the optimized Ar-etching time is determined to be about 30 min.

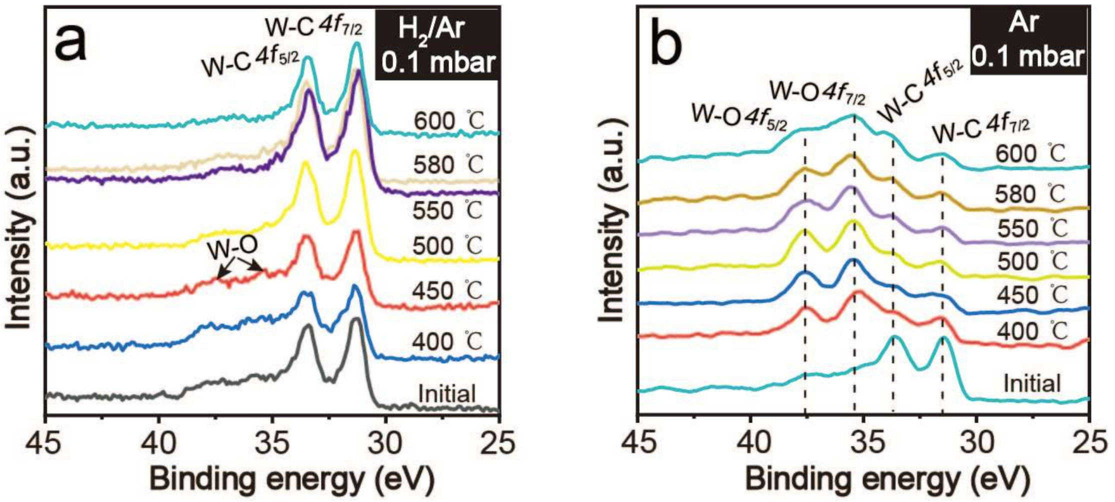


**Supplementary Figure 10.** The subtle change of electronic structure of tungsten atom treated by the increasing temperature under different atmosphere. W *4f* core-level XPS spectra for tungsten species treated in the temperature range of 400 - 600 °C under (a) Ar/$H_2$ (0.1 mbar) and (b) pure Ar (0.1 mbar) atmosphere. The total pressure of Ar/$H_2$ mixture is 0.1 mbar, and the partial pressure of Ar (90%) and $H_2$ (10%) was controlled by two independent leak valves.

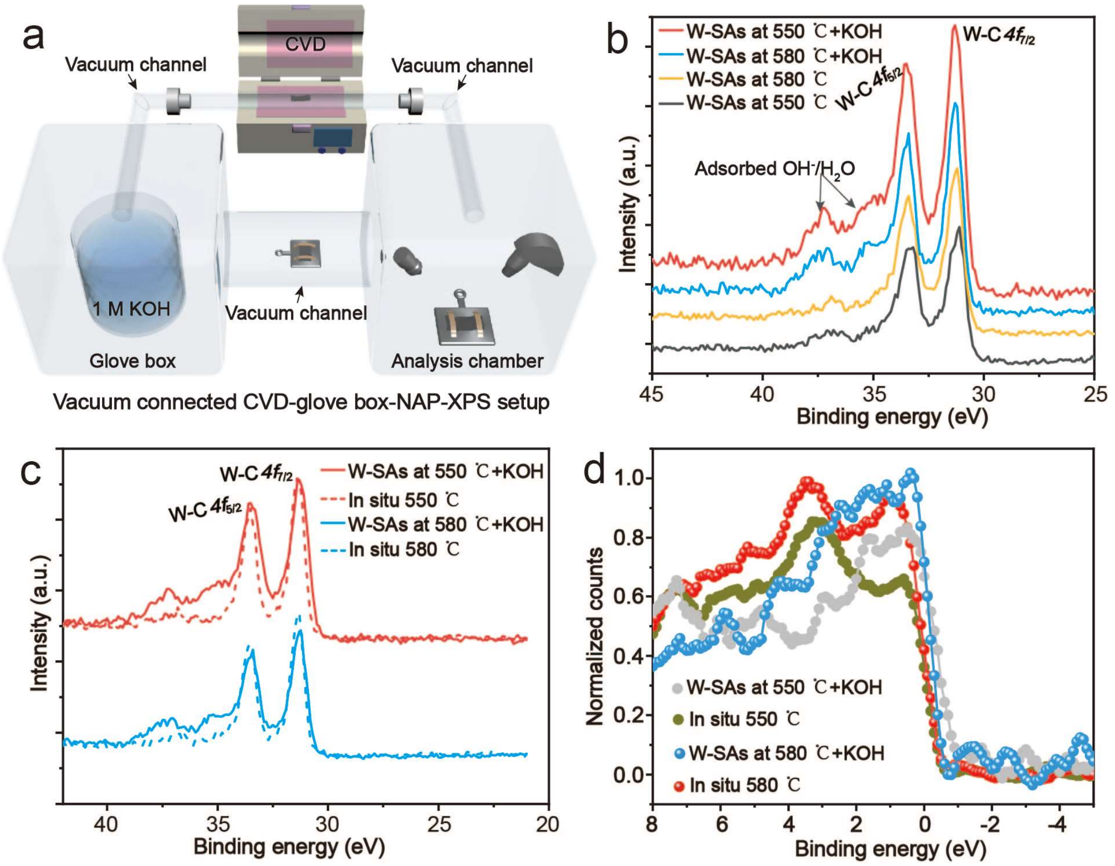


**Supplementary Figure 11.** The subtle change of electronic structure of tungsten

species treated by different temperature using the vacuum-connected CVD-glove box-NAP-XPS setup. (a) Schematic illustration of the vacuum-connected CVD-glove box-NAP-XPS setup. (b) W *4f* core-level XPS spectra of W-SAs annealed at 550 and 580 °C, and second-treatment by 1 M KOH solution, respectively. (c) Comparison of (c) W *4f* XPS and (d) valence band after thermal (550 and 580 °C) and alkaline treatments, and previously collected signals by in situ variable-temperature NAP-XPS measurements.

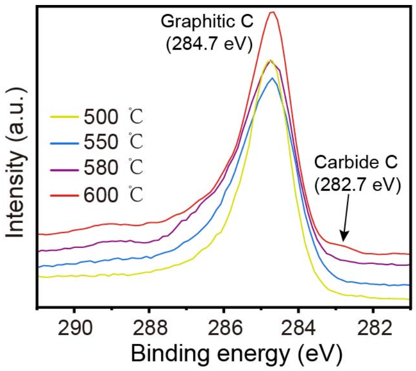


**Supplementary Figure 12**. C *1s* core-level XPS spectra of tungsten species collected at 500, 550, 580, 590 °C ($H_2$, 0.1 mbar). An obvious carbide C *1s* signal at about 282.7 eV can be seen in the spectrum of tungsten species treated at 600 °C, indicating the formation of large-sized WC NPs.

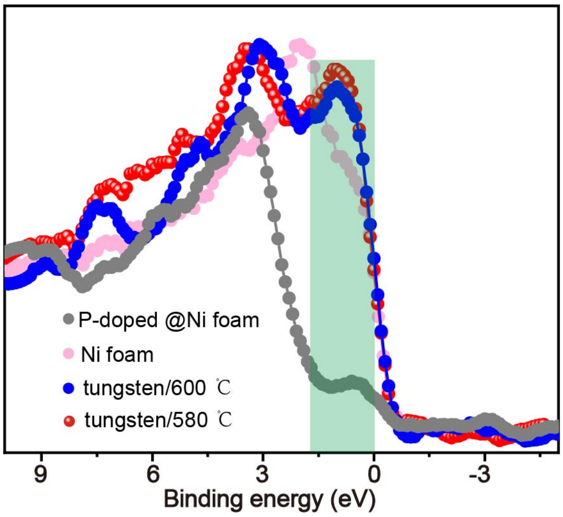


**Supplementary Figure 13**. Valence band of bare Ni foam and that coated with pure P-doped carbon. The intensity of the green-shaded region near the Fermi level is very sensitive to the electronic structure of the sample, hence, compared to those of the

bare Ni foam and C@Ni foam, the stronger signals of the W-SAs treated at 580 and 600 °C can be attributed to the aggregation of W atoms rather than the substrates. The valence band profiles of the reference samples (bare Ni foam and P-doped C@Ni foam) were obtained in the analysis chamber of NAP-XPS system ($5x10^{-9}$ mbar), the valence band profiles of tungsten species at 550 and 580 °C were directly extracted from Fig. 2c.

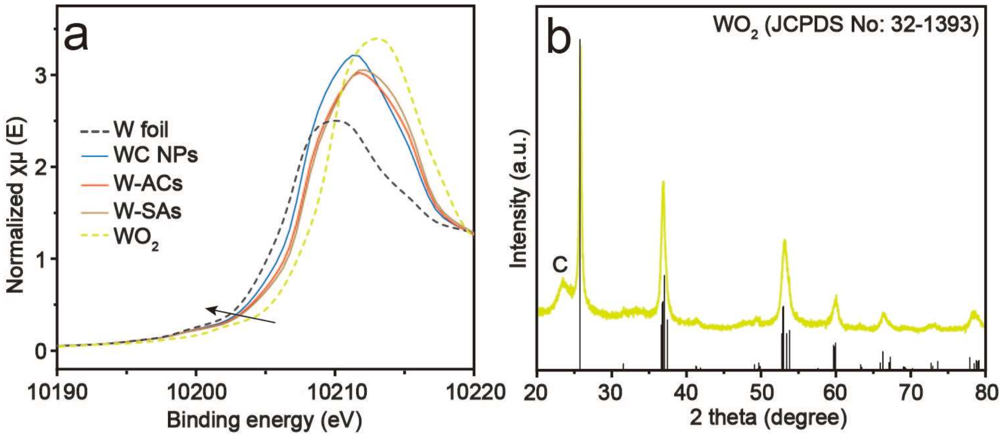


**Supplementary Figure 14**. The determination of valence states of the as-obtained tungsten species using W $L_3$-edge XANES spectra with W foil and $WO_2$ as the references. (a) X-ray absorption near-edge structure (XANES) spectra of W-SAs, W-ACs, WC NPs, and the reference sample of W foil and $WO_2$ powder. (b) XRD pattern of the as-obtained $WO_2$ powder with metallic character.

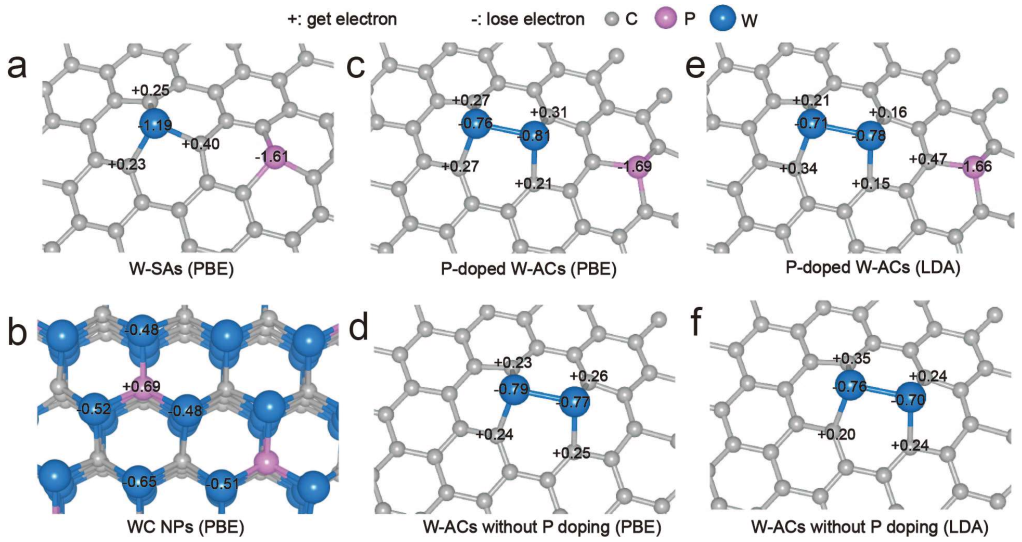


**Supplementary Figure 15**. Bader charge analysis of the as-obtained tungsten species. Changes in Bader charges of W atom in (a) W-SAs, (b) WC NPs, (c, e) P-doped

W-ACs, and (d, f) W-ACs without P doping, in which the values of Bader charge in (a-d) were calculated by the generalized gradient approach (GGA) of the Perdew-Burke-Ernzerhof (PBE) functional[1], whereas another generalized functional of local density approximation (LDA) was used to calculate the ones in (e and f)[2]. Here, C, P, and W atoms are indicated by gray, pink, and blue spheres, respectively.

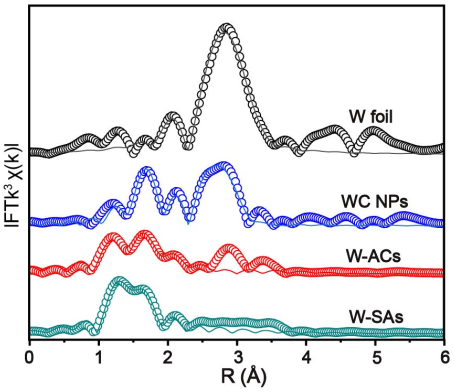


**Supplementary Figure 16**. The characterization of Fourier-transformed extended X-ray absorption fine structure (FT-EXAFS) spectra. W $L_3$-edge FT-EXAFS in R-space for W-SAs, W-ACs, WC NPs, and the reference W foil, where the solid line is the corresponding fitting curve.

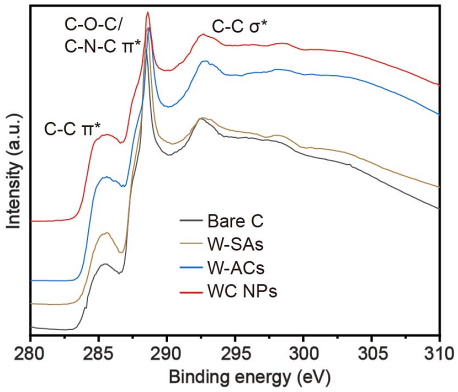


**Supplementary Figure 17**. The characterization of C K-edge near edge X-ray absorption fine structure (C K-edge NEXAFS) spectra. C K-edge NEXAFS spectra of bare C, W-SAs, W-ACs, and WC NPs.

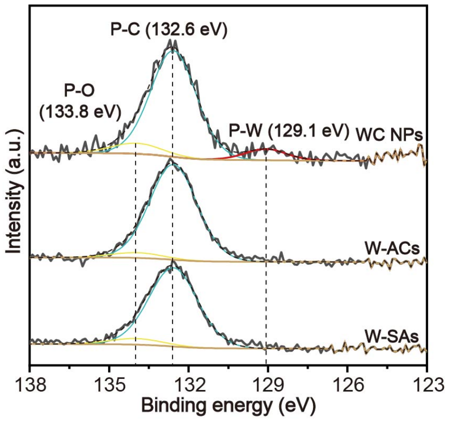


**Supplementary Figure 18**. The characterization of P *2p* core-level XPS spectra. P *2p* XPS spectra of W-SAs, W-ACs, and WC NPs. WC NPs exhibit an additional P-W signal at the binding energy of 129.1 eV, which is almost absent in W-SAs and W-ACs, suggesting that tungsten atoms in W-SAs and W-ACs are not directly coordinated with P atoms in the carbon matrix.

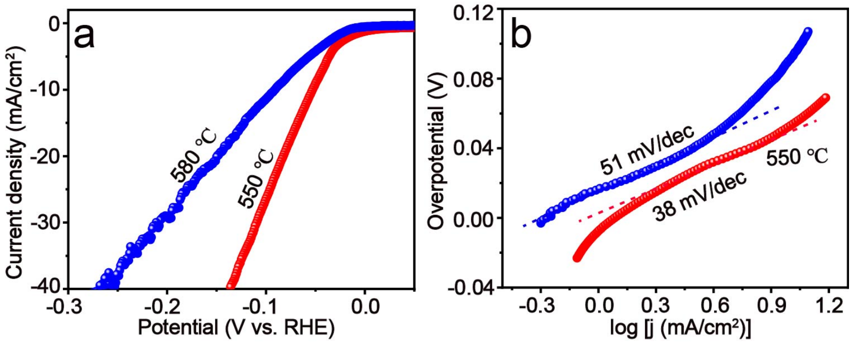


**Supplementary Figure 19**. Alkaline HER performance of tungsten species collected at 550 and 580 °C. (a) LSV curves and (b) Tafel plots of tungsten species synthesized at 550 and 580 °C, respectively.

The tungsten species obtained at 580 °C exhibited a markedly inferior alkaline HER activity in comparison with that of the sample at 550 °C with higher overpotential at 10 mA/cm$^2$ (91 mV) and larger Tafel slope (51 mV/dec), indicating the high-temperature induced graphitic carbon layer on tungsten species has an adverse effect on the electrocatalytic performance.

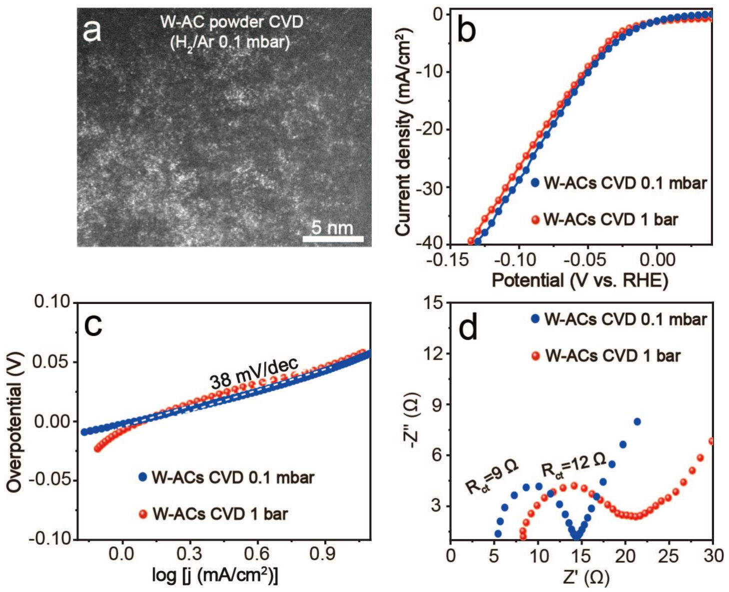


**Supplementary Figure 20**. Alkaline HER performance of W-ACs synthesized at different pressure. (a) HAADF-STEM image, (b) Polarization curves, (c) Tafel plots, and (d) Nyquist plots of W-ACs obtained under Ar/$H_2$ atmosphere at a pressure of 0.1 mbar and 1 bar, respectively.

The values of overpotential ($\eta_{10}$), Tafel slope, and charge transfer resistance ($R_{ct}$) on W-AC powder synthesized at a pressure of 0.1 mbar were determined to be as low as 49 mV, 38 mV/dec, and 9 Ω, respectively, demonstrating its excellent alkaline HER activity

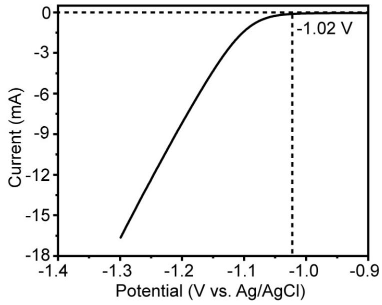


**Supplementary Figure 21**. Calibrating the Ag/AgCl electrode to the reversible hydrogen electrode (RHE). The calibration process was conducted in a standard three-electrode setup under 1.0 M $H_2$-saturated KOH electrolyte with polished Pt wire, carbon rod and Ag/AgCl electrode as the working, counter, and reference electrodes, respectively. Linear scanning voltammetry (LSV) was carried out from -0.9 to -1.3 V at a scanning rate of 5 mV/dec, and the potential at which detached the zero current

point was taken to be the thermodynamic potential (vs. Ag/AgCl) for the hydrogen electrode reaction. The calibrating potential was at -1.02 V in 1.0 M KOH solution; thus, calibration process was proceeded in accordance with the equation: $E_{(RHE)}=E_{(Ag/AgCl)}+1.02$ V.

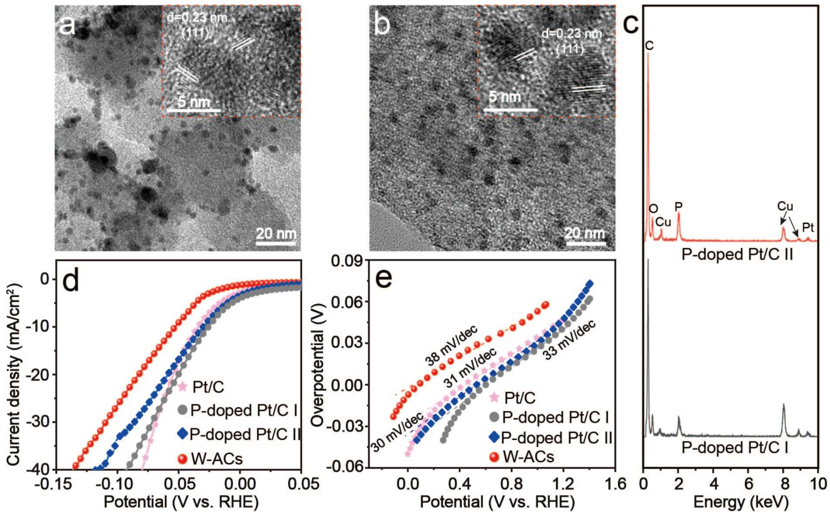


**Supplementary Figure 22**. Morphological observation and alkaline HER performance of different P-doped Pt/C materials. TEM images of (a) P-doped Pt/C I and (b) P-doped Pt/C II, with the corresponding HRTEM images in the insets. (c) The EDX spectrum of P-doped Pt/C I and P-doped Pt/C II, where the C, O, Pt, and P are from the materials, whereas the Cu element originates from the copper grid. (d) LSV curves and (e) Tafel plots of W-ACs, fresh commercial Pt/C, P-doped Pt/C I, and P-doped Pt/C II. The LSV curves of P-doped Pt/C I and P-doped Pt/C II were measured in 1 M KOH electrolyte, in which P-doped Pt/C I exhibited more superior alkaline HER activity ($\eta_{10}$=25 mV) than those of P-doped Pt/C II ($\eta_{10}$=30 mV) and commercial Pt/C ($\eta_{10}$=35 mV).

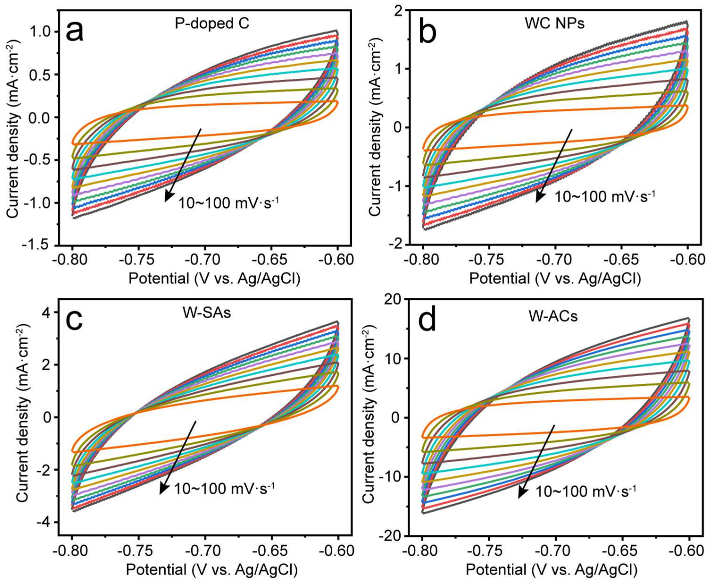


**Supplementary Figure 23**. Cyclic voltammetry (CV) measurements. (a) P doped C, (b) WC NPs, (c) W-SAs, and (d) W-ACs in the voltage window from -0.8 to -0.6 V vs. Ag/AgCl at various scan rates (10-100 mV/s) in alkaline electrolyte.

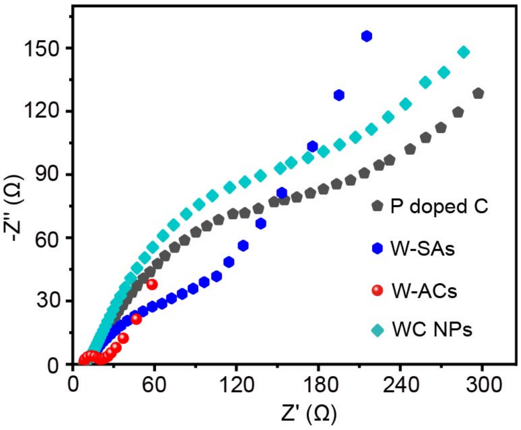


**Supplementary Figure 24**. The characterization of charge transfer resistance. Nyquist plots of P doped C, W-SAs, W-ACs, and WC NPs. W-ACs exhibit a small semicircular diameter as low as 12 Ω, which is much smaller than those of W-SAs and WC NPs, indicating W-ACs possess the fastest charge transfer kinetics.

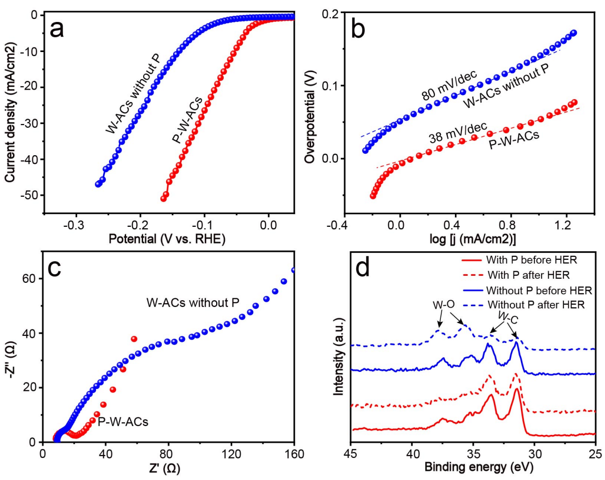


**Supplementary Figure 25**. Alkaline HER performance of W-ACs with and without P doping. (a) LSV curves, (b) Tafel plots and (c) Nyquist plots of W-ACs with and without P dopant. (d) The corresponding W *4f* core-level XPS spectra of samples before and after HER process.

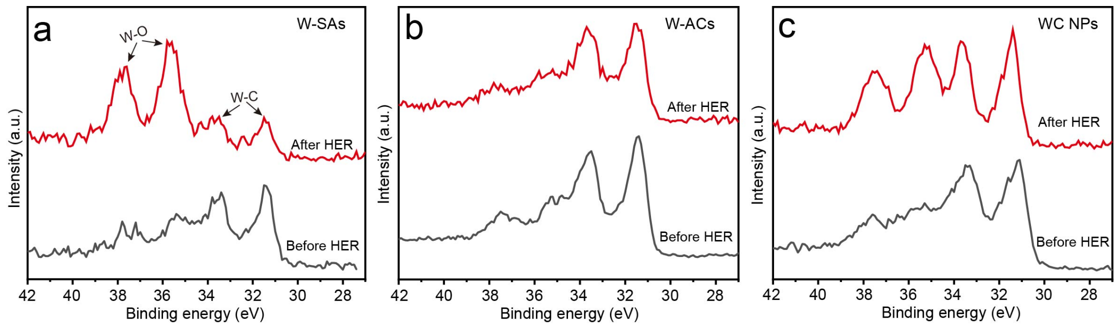


**Supplementary Figure 26**. Surface bonding configurations of different tungsten species before and after HER process. W *4f* core-level XPS spectra of (a) W-SAs, (b) W-ACs and (c) WC NPs. As can be seen, both W-SAs and WC NPs exhibited strong W-O peaks after alkaline HER process. In particular, one could see the markedly decreased W-C signals in W-SAs, indicating the W-SAs have suffered from serious oxidation problems after alkaline HER process, whereas the intrinsic W-C signals without obviously enhanced W-O peaks were detected on W-ACs, highlighting the improved structural robustness of W-ACs in alkaline electrolyte with respect to the W-SA counterparts.

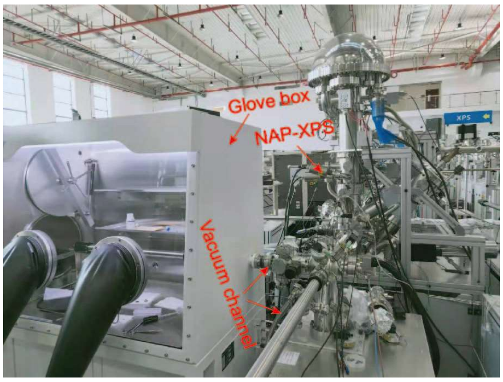


**Supplementary Figure 27**. The display of quasi in situ alkaline HER setup. Photo of the vacuum-connected glove box and NAP-XPS system, where the vacuum connection of glove box and NAP-XPS setup is achieved by vacuum channels, and the electrochemically treated sample can be transferred to the analysis chamber of NAP-XPS without air interference.

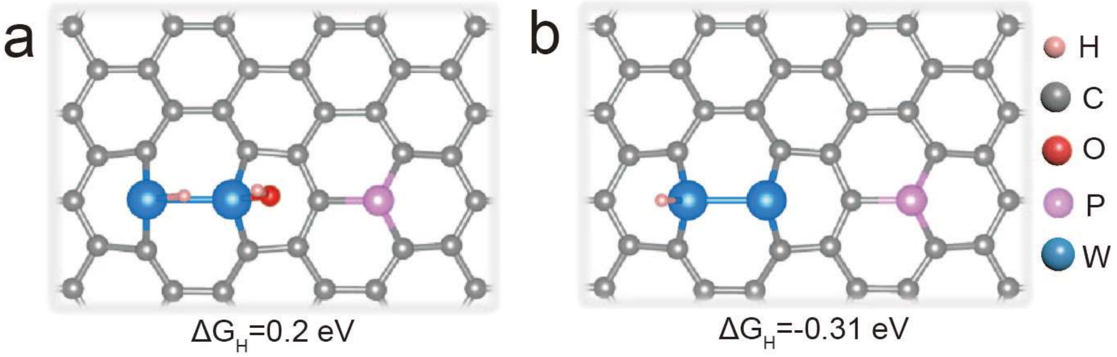


**Supplementary Figure 28**. Hydrogen adsorption free energy of the H* intermediates ($\Delta G_H$) under different conditions. (a) OH* adsorbed on the vicinity of W site. (b) OH* desorbed from the neighboring W site. The presence of OH* intermediate weakens the adsorption strength of the neighboring W-H bonds, yielding $\Delta G_H$ values of up to 0.2 eV, which discourages the formation of the H* intermediates. Here, H, C, O, P, and W atoms are indicated by orange, gray, red, pink, and blue spheres, respectively.

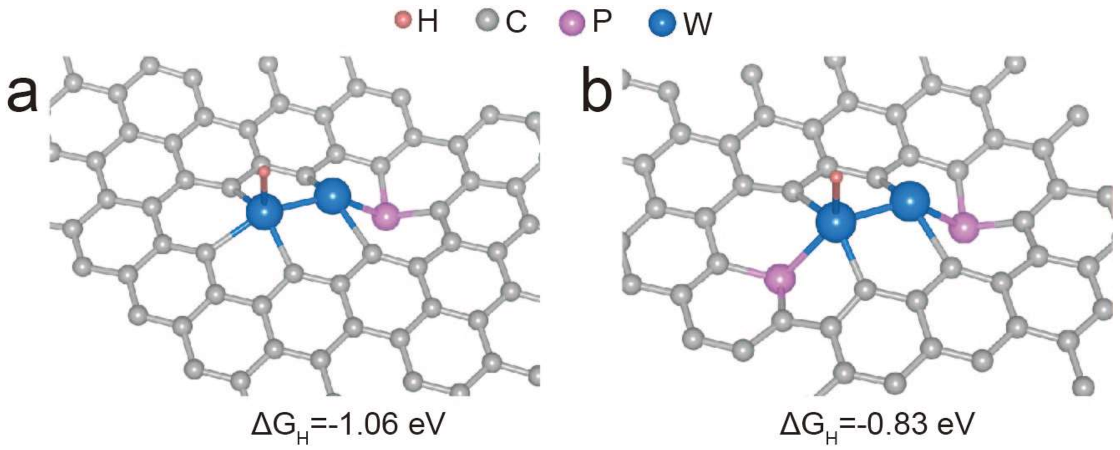


**Supplementary Figure 29**. Geometries of P-doped W-ACs (P directly bonded to W atoms) and the corresponding adsorption free energies ($\Delta G_H$) of possible active sites for H* adsorption. (a) One P atom bonded to W-W dual-atoms. (b) Two P atoms bonded to W-W dual-atoms. Here, H, C, P, and W atoms are indicated by orange, gray, pink, and blue spheres, respectively. The DFT models of one and two P atoms directly bonded to the W-W structures were established. As can be seen, large distortions of the carbon substrate were observed, moreover, the corresponding adsorption free energies (ΔGH) of H* intermediate were calculated to be as high as -1.06 and -0.83 eV, respectively, suggesting they indeed afford inferior HER activities.

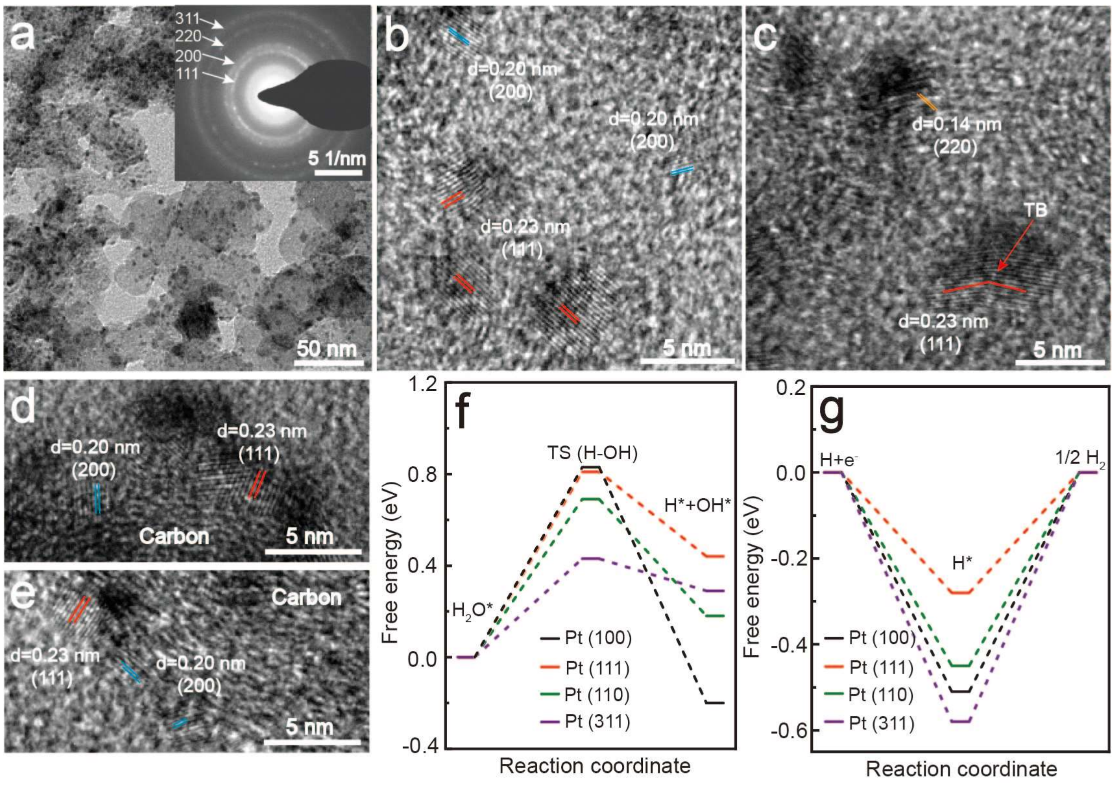


**Supplementary Figure 30**. Morphological observation and DFT calculated alkaline HER activity of Pt. (a) Low TEM image of Pt NPs on the carbon substrates with the corresponding SAED pattern in the inset. (b, c, d, e) HRTEM images of the lattice fringes in Pt, with the twin boundary indicated by the red arrow in (c). The free energy diagrams for (f) water dissociation and (g) hydrogen desorption steps on (111), (100), (110), and (311) facets of Pt.

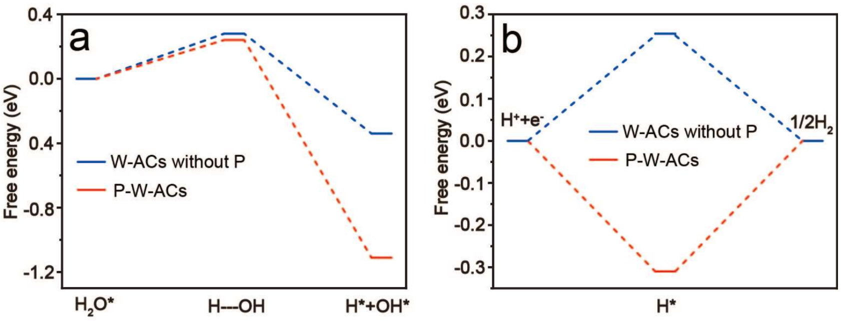


**Supplementary Figure 31**. The free energy diagrams of W-ACs with and without P doping catalyzed alkaline HER process. (a) water dissociation step. (b) Hydrogen desorption step.

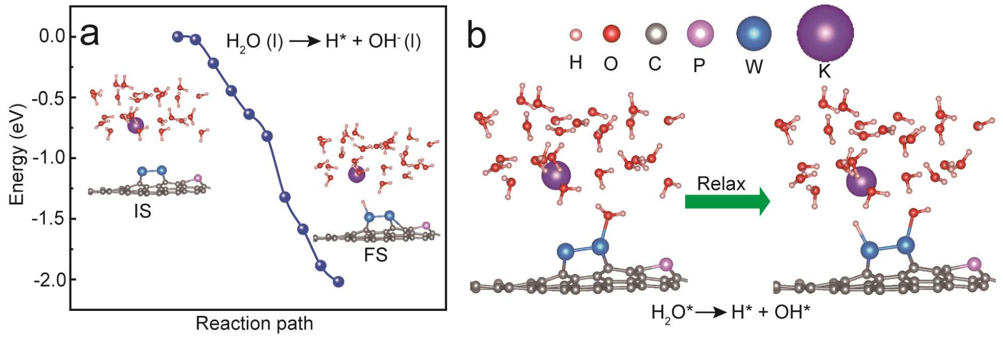


**Supplementary Figure 32.** The analysis of water dissociation step using DFT calculations. The cleavage of H-OH bonds on W-W dual sites in accordance with reaction pathways of (a) $H_2O$ (l) + $e^-$→H* + $OH^-$ and (b) $H_2O$*→H*+OH*, where water-layer model containing metal cation was established for calculating the electrochemical barriers of above-mentioned reaction pathways.

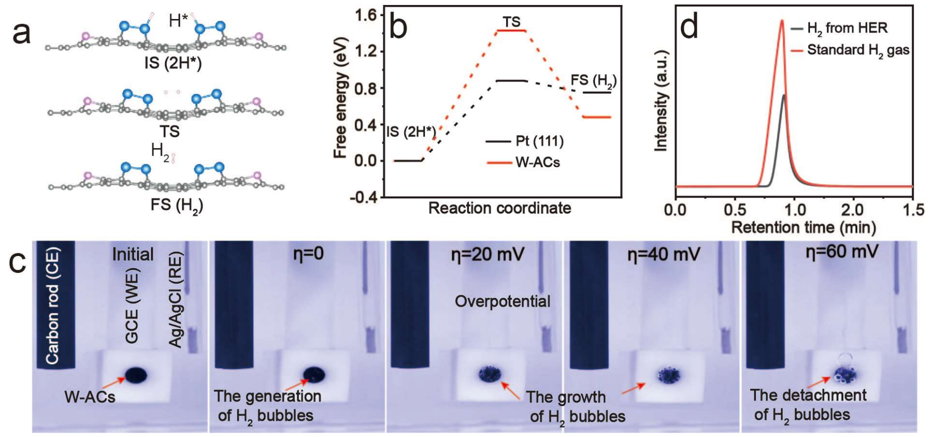


**Supplementary Figure 33.** The analysis of the combination of two H* intermediates into a $H_2$ molecular and the observation of hydrogen generation at different overpotentials. (a) The H* intermediates (IS), transition states (TS), and products of $H_2$ desorption on W-ACs (FS), where H, C, P, and W atoms are indicated by orange, gray, pink, and blue spheres, respectively. (b) The calculated free energy diagram for molecule $H_2$ formation from adsorbed H* based on the calculated models of W-W dimer and Pt (111). (c) Optical images showing the generation and detachment of $H_2$ bubbles under the increasing overpotentials (η). (d) The component analysis of gas bubbles detached from W-ACs catalyst by gas chromatography. In order to directly display the high activity of hydrogen production using the synthesized W-ACs catalyst, a video recording the formation, growth and detachment of electrogenerated hydrogen gas bubbles at the surface of W-ACs at low potentials has

been provided. The product gas was collected for further gas chromatographic analysis, evidencing that the component of the as-obtained gas was $H_2$.

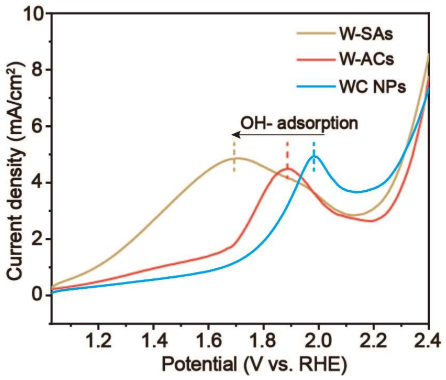


**Supplementary Figure 34**. The evaluation of binding affinity of $OH^-$ on different tungsten species. Oxidative linear sweep voltammetry (LSV) of W-SAs, W-ACs, and WC NPs in 1 M KOH electrolyte.

## Supplementary Tables

**Supplementary Table 1.** The values of the fitting parameters (peak position) of the W *4f* core-level XPS spectra in Fig. 2b, where the 'initial' represents the profile collected at room temperature. The XPS spectra were analyzed and deconvoluted using the Gaussian-Lorentzian sum function with a 30 % Gaussian-Lorentzian value to optimize the spectra, and a Shirley background correction was carried out to subtract the background noise.

| Peak | W-C | | W-C(O) | | W-O | |
|---|---|---|---|---|---|---|
| T (°C) | W $4f_{7/2}$ (eV) | W $4f_{5/2}$ (eV) | W $4f_{7/2}$ (eV) | W $4f_{5/2}$ (eV) | W $4f_{7/2}$ (eV) | W $4f_{5/2}$ (eV) |
| Initial | 31.5 | 33.6 | 32.6 | 34.7 | 35.8 | 37.9 |
| 400 | 31.5 | 33.6 | 32.6 | 34.7 | 35.3 | 37.4 |
| 450 | 31.5 | 33.6 | 32.5 | 34.6 | 35.2 | 37.3 |
| 500 | 31.5 | 33.6 | 32.5 | 34.6 | 35.2 | 37.3 |
| 550 | 31.5 | 33.6 | -- | -- | 35.2 | 37.3 |
| 580 | 31.5 | 33.6 | -- | -- | 35.2 | 37.3 |
| 600 | 31.5 | 33.6 | -- | -- | 35.2 | 37.3 |

**Supplementary Table 2.** The values of the fitting parameters (intensity) of the W *4f* core-level XPS spectra in Fig. 2b (intensity ratio: $I_{W4f5/2}$ : $I_{W4f7/2}$=0.75).

| Peak | W-C | | W-C(O) | | W-O | |
|---|---|---|---|---|---|---|
| T (°C) | W $4f_{7/2}$ | W $4f_{5/2}$ | W $4f_{7/2}$ | W $4f_{5/2}$ | W $4f_{7/2}$ | W $4f_{5/2}$ |
| Initial | 224.5 | 168.4 | 70.3 | 52.7 | 100.5 | 75.3 |
| 400 | 216.2 | 162.2 | 56.5 | 42.4 | 54.4 | 40.8 |
| 450 | 284.3 | 213.2 | 58.6 | 43.9 | 71.1 | 53.3 |
| 500 | 379.1 | 284.3 | 50.1 | 37.6 | 41.8 | 31.3 |
| 550 | 426.7 | 320.1 | -- | -- | 37.1 | 27.8 |
| 580 | 350.6 | 262.9 | -- | -- | 32.7 | 24.5 |
| 600 | 304.7 | 228.5 | -- | -- | 21.4 | 16.1 |

**Supplementary Table 3.** The values of the fitting parameters (full width at half maxima, FWHM) of the W *4f* core-level XPS spectra in Fig. 2b.

| Peak | W-C | | W-C(O) | | W-O | |
|---|---|---|---|---|---|---|
| T (°C) | W $4f_{7/2}$ (eV) | W $4f_{5/2}$ (eV) | W $4f_{7/2}$ (eV) | W $4f_{5/2}$ (eV) | W $4f_{7/2}$ (eV) | W $4f_{5/2}$ (eV) |
| Initial | 0.96 | 0.96 | 0.97 | 0.97 | 1.15 | 1.15 |
| 400 | 0.96 | 0.96 | 0.97 | 0.97 | 1.15 | 1.15 |
| 450 | 0.96 | 0.96 | 0.97 | 0.97 | 1.15 | 1.15 |
| 500 | 0.91 | 0.91 | 0.97 | 0.97 | 1.15 | 1.15 |
| 550 | 0.88 | 0.88 | -- | -- | 1.18 | 1.18 |
| 580 | 0.88 | 0.88 | -- | -- | 1.18 | 1.18 |
| 600 | 0.88 | 0.88 | -- | -- | 1.18 | 1.18 |

**Supplementary Table 4**. Structural parameters for different W-based samples extracted from the W $L_3$-edge EXAFS fitting. Here, 'Shell' is the bonding type, 'CN' is the coordination number of the W atom, 'R' (Å) is the fitting bonding length, '$\sigma^2$' represents the Debye-Waller factor, and the R-factor is used to evaluate the quality of the fitting results.

| Sample | Shell | CN | R | $\sigma^2$ | R-factor |
|---|---|---|---|---|---|
| W foil | W-W | 8 | 2.78 | 0.00102 | 0.0059 |
| | W-W | 6 | 3.16 | 0.00058 | |
| WC | W-C | 6 | 2.12 | 0.00503 | 0.0259 |
| | W-W | 2 | 2.81 | 0.001 | |
| | W-W | 6 | 2.89 | 0.00434 | |
| W-SAs | W-C | 1.0 | 1.72 | 0.00337 | 0.0141 |
| | W-C | 2.1 | 2.16 | 0.00580 | |
| W-ACs | W-C | 1.8 | 2.17 | 0.00987 | 0.202 |
| | W-W | 1.1 | 2.15 | 0.00340 | |

**Supplementary Table 5.** The values of the fitting parameters (peak position) of the W 4f core-level XPS spectra in Fig. 4b, where the 'initial' represents the profile collected under the open circuit potential condition (only soaked in KOH electrolyte), the unit of the applied potential is Volts (V). The XPS spectra were analyzed and deconvoluted

using the Gaussian-Lorentzian sum function with a 30 % Gaussian-Lorentzian value to optimize the spectra, and a Shirley background correction was carried out to subtract the background noise. Where the ‘initial’ represents the profile collected at room temperature.

| Peak | W-C | | W-C(O) | | W-O | |
|---|---|---|---|---|---|---|
| State | W $4f_{7/2}$ (eV) | W $4f_{5/2}$ (eV) | W $4f_{7/2}$ (eV) | W $4f_{5/2}$ (eV) | W $4f_{7/2}$ (eV) | W $4f_{5/2}$ (eV) |
| Initial | 31.5 | 33.6 | 32.5 | 34.6 | 35.2 | 37.3 |
| 0.00 | 31.5 | 33.6 | 32.5 | 34.6 | 35.6 | 37.7 |
| -0.02 | 31.5 | 33.6 | 32.5 | 34.6 | 35.2 | 37.3 |
| -0.04 | 31.5 | 33.6 | 32.5 | 34.6 | 35.2 | 37.3 |
| -0.06 | 31.5 | 33.6 | 32.5 | 34.6 | 35.2 | 37.3 |

**Supplementary Table 6.** The values of the fitting parameters (intensity) of the W *4f* core-level XPS spectra in Fig. 4b (intensity ratio: $I_{W4f_{5/2}}$ : $I_{W4f_{7/2}}$=0.75).

| Peak | W-C | | W-C(O) | | W-O | |
|---|---|---|---|---|---|---|
| State | $W4f_{7/2}$ | $W4f_{5/2}$ | $W4f_{7/2}$ | $W4f_{5/2}$ | $W4f_{7/2}$ | $W4f_{5/2}$ |
| Initial | 418.3 | 313.7 | 114.1 | 85.6 | 156.9 | 117.7 |
| 0.00 | 402.2 | 301.7 | 180.2 | 135.2 | 266.2 | 199.7 |
| -0.02 | 405.4 | 304.1 | 123.6 | 92.7 | 133.1 | 99.8 |
| -0.04 | 406.2 | 304.7 | 103.4 | 77.6 | 118.8 | 89.1 |
| -0.06 | 401,3 | 300.9 | 102,4 | 76.8 | 127.1 | 95.3 |

**Supplementary Table 7.** The values of the fitting parameters (full width at half maxima, FWHM) of the W *4f* core-level XPS spectra in Fig. 4b.

| Peak | W-C | | W-C(O) | | W-O | |
|---|---|---|---|---|---|---|
| State | W $4f_{7/2}$ (eV) | W $4f_{5/2}$ (eV) | W $4f_{7/2}$ (eV) | W $4f_{5/2}$ (eV) | W $4f_{7/2}$ (eV) | W $4f_{5/2}$ (eV) |
| Initial | 0.92 | 0.92 | 0.93 | 0.93 | 1.15 | 1.15 |
| 0.00 | 0.92 | 0.92 | 0.93 | 0.93 | 1.38 | 1.38 |
| -0.02 | 0.92 | 0.92 | 0.93 | 0.93 | 1.38 | 1.38 |
| -0.04 | 0.92 | 0.92 | 0.93 | 0.93 | 1.38 | 1.38 |
| -0.06 | 0.92 | 0.92 | 0.93 | 0.93 | 1.41 | 1.41 |

**Supplementary Table 8.** The values of the fitting parameters (peak position, intensity, full width at half maxima (FWHM)) of the O *1s* core-level XPS spectra in Fig. 4c. Where the 'initial' represents the profile collected under the open circuit potential condition (only soaked in KOH electrolyte), the unit of the applied potential is Volts (V).

| Peak | W-O | | | C-O | | |
|---|---|---|---|---|---|---|
| State | Position | Intensity | FHWM | Position | Intensity | FHWM |
| Initial | 530.9 | 517.8 | 1.97 | 531.9 | 229.2 | 2.17 |
| 0.00 | 530.9 | 772.5 | 2.09 | 531.9 | 297.1 | 2.30 |
| -0.02 | 530.9 | 577.1 | 2.01 | 531.9 | 322.6 | 2.21 |
| -0.04 | 530.9 | 560.2 | 2.04 | 531.9 | 237.7 | 2.24 |
| -0.06 | 530.9 | 356.5 | 2.11 | 531.9 | 220.7 | 2.30 |

**Supplementary Table 9**. The calculated activation energy of $H_2O$ ($\Delta G_{H_2O}$), and the adsorption energies of the produced OH* and H* intermediates on the W-SAs, W-ACs, and WC NPs.

| Materials | $\Delta G_{H2O}$ (eV) | $\Delta G_{OH}$ (eV) | $\Delta G_{H}$ (eV) |
|---|---|---|---|
| W-SAs | 0.65 | -1.65 | -0.46 |
| W-ACs | 0.24 | -1.42 | -0.31 |
| WC NPs | 0.41 | -0.53 | -0.61 |

## Supplementary Methods

**Preparation of $WO_2$ reference**: The synthesis of $WO_2$ powder was in accordance with previous report[3]. In brief, commercial $WO_3$ (0.2 g) was dissolved in ethylenediamine (EDA, 15 mL) with continuous stirring for 30 min in a Teflon-lined stainless-steel autoclave (20 mL). Then the reactants were heated at 180 °C for 8 h. After the autoclave cooled down to the room temperature, the resultant white product ($WO_3$-EDA) was collected after centrifugation and washed with deionized water and absolute ethanol. Subsequently, the as-obtained $WO_3$-EDA precursors were pyrolyzed at 700 °C with a heating rate of 1 °C/min for 5h at Ar atmosphere, and the metallic $WO_2$/C sample was synthesized.

**Preparation of different P-doped Pt/C catalysts**: P-doped Pt/C I was obtained by the low-temperature phosphidation of commercial Pt/C[4], whereas P-doped Pt/C II was collected according to the synthetic method of tungsten precursors with $PtCl_2$ regent replacing $Na_2WO_4{\cdot}2H_2O$, in which 3 g of polydopamine (PDA), 3 g of $P_{123}$, 0.5 g of Tris, 0.6 g of $Na_2H_2PO_4{\cdot}2H_2O$, and 0.03 g of $PtCl_2$ were dissolved in distilled water (30 mL) with continuous stirring for 6 h. Then, the Pt precursor was collected by filtration, washed with a mixture of distilled water and ethanol, and then dried at 60 °C. After pyrolysis at 700 °C for 2h under Ar condition, and followed by annealing at Ar/$H_2$ atmosphere for 30 min, P-doped Pt/C II sample was synthesized

**Supplementary Notes**

**Supplementary Note 1: in situ variable-temperature NAP-XPS measurement.**

As shown in Supplementary Fig. 8, the observation of the structural evolution of tungsten species from W-SAs to WC NPs by thermal migration (structure characterization) was carried out in the analysis chamber of NAP-XPS (SPEC NAP-XPS) at $H_2$ atmosphere with a pressure of 0.1 mbar, and the treating temperature was controlled by the laser heating device and the thermocouple equipped. Obviously, it is an in situ NAP-XPS measurement.

**Supplementary Note 2: the determination of optimized Ar-etching time.**

As shown in Supplementary Fig. 9, the W-SAs exhibit a sharp decrease in the concentration of the surface $WO_x$ passivation layer with increase in the Ar-etching time, as indicated by the decreased intensity of the W-O bonds at about 35.3 ($4f_{7/2}$) and 37.8 eV ($4f_{5/2}$). However, longer treating times (35 min) would affect the intrinsic W-C carbide structure. Therefore, the optimized Ar-etching time was determined to be 30 min. It should be noted that the Ar-etching treatment was proceeded in the analysis chamber of NAP-XPS system under Ar atmosphere (5 x $10^{-6}$ mbar), and the W *4f* XPS signals were then recorded to determine the optimized Ar-etching time under vacuum condition (5 x $10^{-9}$ mbar).

**Supplementary Note 3: the preparation process of the powders and the XPS experiment monitor the same chemical processes.**

In order to ensure that the preparation process of the powders and the XPS experiment monitor the same chemical processes, in situ variable-temperature NAP-XPS experiments at Ar/$H_2$ (0.1 mbar) and pure Ar (0.1 mbar) atmosphere have also been conducted, respectively (Supplementary Fig. 10). For the W *4f* XPS signals of the tungsten species collected above 500 °C at Ar/$H_2$ (0.1 mbar) atmosphere (Supplementary Fig. 10a), the W $4f_{7/2}$ (W-C) intensity increased upon heating to 550 °C and decreased when further heating to 580 °C, in which the enhanced intensity of the W-C bond indicated the formation of a high density of tungsten clusters, whereas the simultaneous coating of the graphitic carbon on the formed tungsten

clusters should be responsible for the decreased intensity[5,6]. Similar change of the W *4f* XPS signals has been observed under pure $H_2$ atmosphere (0.1 mbar) in the main text (Fig. 2b). Meanwhile, a small difference between these two experimental results should be pointed out: an obvious decrease of the W-C peak together with the slightly increased W-O signals was observed in the tungsten species treated below 500 °C under Ar/$H_2$ atmosphere (which were invisible under a pure $H_2$ atmosphere), indicating a partial oxidation of the tungsten species below 500 °C, probably originated from the chemisorbed oxygen in the surficial region of carbon substrate that released in the Ar/$H_2$ atmosphere. In contrast, a clear oxidation phenomenon with markedly strong W-O signals was observed on tungsten species from 400 to 600 °C under pure Ar atmosphere (Supplementary Fig. 10b), indicating the tungsten species have bonded to the substrate oxygen atoms without the protection of hydrogen atmosphere, which severely impeded the detection of the intrinsic W-C carbide structures. In comparison with the previous results obtained in pure $H_2$, the above two control experiments confirm the reducing atmosphere provided by $H_2$ gas plays a vital role in observing the structure evolution of tungsten species and identifying the optimized synthesized temperature of W-ACs.

**Supplementary Note 4: whether in situ variable-temperature NAP-XPS measurement is necessary.**

We have treated the W-SA samples at 550 and 580 °C respectively in vacuum-connected CVD tubular furnace at $H_2$ atmosphere, followed by transferring the treated samples to the analysis chamber for XPS measurements via vacuum channels (Supplementary Fig. 11a). As can be seen, the two samples (550 and 580 °C) exhibited relatively weak W-C signals (Supplementary Fig. 11b), suggesting the surface carbide structure was probably affected by the diffused graphitic impurities and small amount of stubborn oxide atoms for the cooled down sample[5,7]. However, an obviously enhanced intensity of W-C peak was detected after alkaline treatment in the vacuum-connected glove box[7,8], in which the W-C carbide signals were extremely similar to those collected by in situ variable-temperature NAP-XPS measurements under the same temperature (Supplementary Fig. 11c), indicating the surface

impurities (noncarbidic carbon, oxygen species) affecting the detection of W-C carbide signals have been removed by alkaline treatment[7,8]. Moreover, the corresponding valence band at the Fermi level (0~2 eV) were also comparable to that observed by in situ variable-temperature NAP-XPS measurements (Supplementary Fig. 11d), demonstrating their Pt-like electronic structure after alkaline treatment. Therefore, comparing the above results obtained in the vacuum-connected CVD tubular furnace and those of in situ variable-temperature NAP-XPS measurements, two important findings should be pointed out: (i) in situ variable-temperature NAP-XPS measurements can be used to directly detect the intrinsic signals of the aggregated tungsten species without any interference due to the protection from reducing atmosphere at high temperature; (ii) the vacuum-connected CVD and NAP-XPS system can clearly reveal the enhanced W-C carbide signals in relevance to the aggregation of W-SAs, while an additional surface cleaning procedure using alkaline treatment should be introduced in glove box to avoid interferences from the surface graphitic carbon and stubborn oxygen species for the cooled down sample, which is obviously more complicated than the in situ variable-temperature NAP-XPS measurements.

**Supplementary Note 5: whether the small difference of Bader charge on W-ACs is valuable for alkaline HER activity.**

Based on the Bader charge analysis in Supplementary Fig. 15, two important findings should be pointed out: (i) the amount of charges on the tungsten atoms in W-ACs and WC NPs transferred to the ligands was lower than that of the W-SAs, and the W-ACs contain tungsten in a moderate oxidation state, which may be beneficial for the alkaline HER process; (ii) compared to the W-ACs without P doping, it is clear that the W atom near P atom suffers larger amount of depleted electrons than the others on the basis of calculations using PBA and LDA functional, indicating the introduction of P atom as a dopant indeed modifies the electronic structures of the W-W dimer. In addition, the small change of above calculated Bader charges was found to be significant for HER activity as presented by previous reports. For example, Zhu et al. reported the $WS_2/MoS_2$ heterostructure for electrocatalytic HER process[9], in

which Bader charge analysis revealed that a charge transfer of 0.014 e from $WS_2$ to $MoS_2$ will inevitably facilitate the overall HER process. Moreover, Huang et al. reported a volcano relationship between the hydrogen adsorption ability (HER activity) and Bader charges carried by Ru11, which demonstrated that the the small change (0.0172 e) of Bader charges in Zn-doped Ru11 can make the HER activity close to the volcano peak in comparison with that of Fe doping[10]. Therefore, our small change (>0.04 e) of Bader charges can also be used to predict the improved alkaline HER activity on P-doped W-ACs.

## Supplementary Discussion

**Supplementary Discussion 1: alkaline HER activities of W-ACs with and without P doping.**

The P-doped W-ACs indeed exhibited more superior alkaline HER activity than that of the sample without P-doping in 1 M KOH electrolyte, as determined by the lower overpotential and Tafel slope (Supplementary Fig. 25a and b). As can be seen, the introduction of P atom into the W-ACs can indeed boost the alkaline HER activities, in which the P-doping could accelerate the charge transfer during the alkaline HER process, due to the highly polarized P-C bond formed between P and C with different electronegativities[11,12], which will serve as an electron transfer bridge to improve the overall conductivity, as determined by the small charge transfer resistance (Supplementary Fig. 25c). Meanwhile, the fast charge transfer can also boost the prior water dissociation step and the accumulation of the electrons in cathode, thus accelerating the desorption of OH* intermediates, as evidenced by the comparison of W *4f* core-level XPS signals collected before and after alkaline HER process on W-ACs with and without P doping (Supplementary Fig. 25d).

**Supplementary Discussion 2: the change of surface configurations of different tungsten species before and after HER process.**

As shown in Supplementary Fig. 26, both W-SAs and WC NPs exhibit strong W-O peaks after alkaline HER process, in particular, one could see the markedly decreased W-C signals in W-SAs, indicating they have suffered from serious oxidation problems because of the corrosion of adsorbed OH* intermediates in the alkaline HER process, whereas the intrinsic W-C signals without obviously enhanced W-O peaks were detected on W-ACs, highlighting the improved structural robustness of W-ACs in alkaline electrolyte when compared to the W-SA and WC counterparts.

**Supplementary Discussion 3: the difference of alkaline HER activities using commercial Pt/C catalyst evaluated by DFT calculations and practical application.**

The commercial Pt/C powder-catalyzed alkaline HER process actually involved the synergistic work of multiple crystalline planes, such as (111), (100), (110), (311) and so forth, as observed by the transmission electron microscopy (TEM). As shown in the low-magnification TEM image, a large amount of Pt NPs with sizes less than 5 nm were uniformly dispersed on the carbon substrates (Supplementary Fig. 30a), and the corresponding selected area electron diffraction (SAED) pattern (inset in Supplementary Fig. 30a) showed the polycrystalline nature of the nanopaticles with the presence of (111), (200), (220), and (311) facets, respectively. The marked d-spacing values of 0.23, 0.20, and 0.14 nm in the HRTEM images are well in agreement with the (111), (200), and (220) planes of Pt NPs, respectively (Supplementary Fig. 30b-e). Accordingly, the energy barriers in water dissociation and hydrogen desorption steps on above observed surfaces were calculated for comparison. The resultant values of water dissociation barriers over (111), (100), (110), and (311) facets of Pt were 0.81, 0.83, 0.69, and 0.43 eV, respectively (Supplementary Fig. 30f), indicating the Pt (311) has the lowest energy barrier in water dissociation step and the energy barrier of Pt in the rate-determining step (water dissociation) has been substantially weakened, which also confirms that the Pt (111) is generally inefficient for the cleavage of H-OH bonds[13,14]. However, the calculation in the subsequent hydrogen desorption step evidenced that Pt (111) takes the fastest hydrogen desorption process with energy barrier as low as 0.28 eV (Supplementary Fig. 30f), which is much lower than those of other crystalline planes (Supplementary Fig. 30g). In addition, another three advantages of commercial Pt/C powders that were neglected by DFT calculations should also be emphasized for boosting alkaline HER activity: (i) more available and highly active sites for Pt-catalyzed HER process could be introduced due to their ultrasmall size (< 5 nm)[7,15]; (ii) the observed twin boundary (TB) of Pt (111) facet can efficiently boost the electrocatalytic activity (Supplementary Fig. 30c), because they can provide more low-coordination atomic sites for alkaline HER process, which are beneficial for electrocatalysis[16]; (iii) the energy barriers of alkaline HER process can be regulated by the synergistic work of carbon layer and encapsulated Pt NPs[17,18]. Therefore, according to the calculated

energy barriers in the two-step alkaline HER process and previous reports, we can conclude that multiple crystal planes, ultrasmall size, twin boundary, and carbon-Pt interface work in synergy to alter the energy barriers for the observed excellent alkaline HER activity on commercial Pt/C catalyst.

**Supplementary Discussion 4: the comparison of $H_2O$ (l) + $e^-$→H* + $OH^-$ (l) and $H_2O$*→H*+OH* reaction pathways.**

The electrochemical barriers of $H_2O$ (l) + $e^-$→H* + $OH^-$ (l) and $H_2O$*→H*+OH* were calculated in accordance with the water-layer model containing metal cation and previously reported step-wise desorption process of adsorbed OH* intermediates[19,20]. As expected, no matter what reaction pathway was followed, no energy barriers were calculated for the cleavage of H-OH bonds on W-W dual sites (Supplementary Fig. 32a and b), indicating W-W dual-sites indeed have strong ability to break H-OH bonds. Then, the produced H* intermediates were adsorbed on the W sites, whereas the split $OH^-$ radicals were directly retained in the electrolyte without desorption process based on the reaction pathway of $H_2O$ (l) + $e^-$→H* + $OH^-$ (l). However, the adsorbed OH* intermediates will be repelled into the electrolyte when increasing applied potentials based on our experimental observations. Note that the realistic electrocatalytic environment is much more complicated than that of the established DFT model, and thereby much more powerful analytic techniques are required for a further comprehension of the catalytic mechanism[21], but our DFT prediction and aforementioned experimental observations have evidenced that the reaction pathway of $H_2O$*→H*+OH* and subsequent desorption of OH* should also be reasonable.

**Supplementary Discussion 5: the energy barriers of $H_2$ formation from adsorbed H* (activation energy) on W-ACs and Pt (111).**

The energy barriers of $H_2$ formation from adsorbed H* (activation energy) on W-ACs and Pt (111) (the optimal facet of hydrogen desorption) have been calculated. As shown in Supplementary Fig. 33a, a pair of W-W dimer were constructed to calculate the energy barrier of molecule $H_2$ formation, in which the molecule $H_2$ was generated between the dimer rather than the W atoms, because the W sites have strong

dissociation ability of molecule $H_2$[22]. As a result, the energy barrier was calculated to be approximately 1.43 eV (Supplementary Fig. 33b), which is higher than that of Pt (111) (0.88 eV)[23]. Therefore, combining previous small energy barriers in water dissociation (0.24 eV) and H* desorption (-0.31 eV) on W-ACs, we can conclude that the relatively high energy barrier in molecule $H_2$ formation should be further regulated on W-ACs in our future work.

**Supplementary References**


1. Perdew, J. P., Burke, K., Ernzerhof, M. Generalized gradient approximation made simple. *Phys. Rev. Lett.* **77**, 3865-3868 (1996).
2. Pirovano, M. V. G., Silva, J. L. F. D., Sauer, J. Density-functional calculations of the structure of near-surface oxygen vacancies and electron localization on $CeO_2$(111). *Phys. Rev. Lett.* **102**, 026101 (2009).
3. Wu, R., Zhang, J. F. et al. Metallic $WO_2$-carbon mesoporous nanowires as highly efficient electrocatalysts for hydrogen evolution reaction. *J. Am. Chem. Soc.* **137**, 6983-6986 (2015).
4. Zhang, Z, Y., Liu, S. S. et al. Fiber-based multifunctional nickel phosphide electrodes for flexible energy conversion and storage. *J. Mater. Chem. A*, **4**, 9691-9699 (2016).
5. Hunt, S. T., Nimmanwudipong, T., Leshkov, Y. R. Engineering non-sintered, metal-terminated tungsten carbide nanoparticles for catalysis. *Angew. Chem. Int. Ed.* **53**, 5131-5136 (2014).
6. Ko, Y. J., Cho, J. M. et al. Tungsten carbide nanowalls as electrocatalyst for hydrogen evolution reaction: New approach to durability issue. *Appl. Catal. B: Environ*, **203**, 684-691 (2017).
7. Xu, Y. T., Xiao, X. F. et al. Cage-confinement pyrolysis route to ultrasmall tungsten carbide nanoparticles for efficient electrocatalytic hydrogen evolution. *J. Am. Chem. Soc.* **139**, 5285-5288 (2017).
8. Yang, X. F., Kimmel, Y. C. et al. Activation of tungsten carbide catalysts by use of an oxygen plasma pretreatment. *ACS Catal.* **2**, 765-769 (2012).
9. Li, H. L., Yu, K. et al. Enhanced hydrogen evolution performance of ultra thin nanoslice/nanopetal structured $XS_2$ (X=W, Mo): From experiment to theory. *J. Appl. Phys.* **120**, 024301 (2016).
10. Ren, M. M., Guo, X. Y. et al. Transition metal atoms (Fe, Co, Ni, Cu, Zn) doped RuIr surface for the hydrogen evolution reaction: A first-principles study. *Appl. Surf. Sci.* **556**, 149801 (2021).
11. Zheng, Y. R., Wu, P. et al. Doping-induced structural phase transition in cobalt diselenide enables enhanced hydrogen evolution catalysis. *Nat. Commun.* **9**, 2533 (2018).
12. Liu, H., Liu, Y. Y. et al. Surface phosphorus-induced CoO coupling to monolithic carbon for efficient air electrode of quasi-solid-state Zn-air batteries. *Adv. Sci.* **8**, 2101314 (2021).
13. Li, G. W., Fu, C, G. et al. Dirac nodal arc semimetal $PtSn_4$: An ideal platform for understanding surface properties and catalysis for hydrogen evolution. *Angew. Chem. Int. Ed.* **58**, 13107-13112 (2019).
14. Chen, L. W., Guo, X. et al. Structurally ordered intermetallic $Ir_3V$ electrocatalysts for alkaline hydrogen evolution reaction. *Nano Energy* **81**, 105636 (2021).
15. Gong, Q. F. Wang, Y. et al. Ultrasmall and phase-pure $W_2C$ nanoparticles for efficient electrocatalytic and photoelectrochemical hydrogen evolution. *Nat. Commun.* **7**, 13216 (2016).

16. Liu, J. W., Niu, W. X. et al. Selective epitaxial growth of Rh nanorods on 2H/fcc heterophase Au nanosheets to Form 1D/2D Rh-Au heterostructures for highly efficient hydrogen evolution. *J. Am. Chem. Soc.* **143**, 4387-4396 (2021).
17. Deng, J., Ren, P. J. et al. Enhanced electron penetration through an ultrathin graphene layer for highly efficient catalysis of the hydrogen evolution reaction. *Angew. Chem. Int. Ed.* **54**, 2100-2104 (2015).
18. Tu, Y. C., Ren, P. J. et al. Structural and electronic optimization of graphene encapsulating binary metal for highly efficient water oxidation. *Nano energy* **52**, 494-500 (2018).
19. Lamoureux, P. S., Singh, A. R., Chan, K. pH effects on hydrogen evolution and oxidation over Pt(111): insights from first-principles. *ACS Catalysis* **9**, 6194-6201 (2019).
20. Chan, K., Nørskov, J. K. Electrochemical barriers made simple. *J. Phys. Chem. Lett.* **6**, 2663-2668 (2015).
21. *Catalysis as it goes*. *Nat Catal* **1**, 165-166 (2018).
22. Duan, Y., Yu, Z. Y. et al. Bimetallic nickel-molybdenum/tungsten nanoalloys for high-efficiency hydrogen oxidation catalysis in alkaline electrolytes. *Nat. Commun.* **11**, 4789 (2020).
23. Yu, C. M., Wang, F. et al. $H_2$ thermal desorption spectra on Pt(111): A density functional theory and kinetic monte carlo simulation study. *Catalysts*, **8**, 450 (2018).
