# Supplementary material for: Thermal migration towards constructing W-W dual-sites for boosted alkaline hydrogen evolution reaction
Source: Nat Commun. 2022 Feb 9;13:763. doi: 10.1038/s41467-022-28413-6 (PMC8828749; doi:10.1038/s41467-022-28413-6)
Supplement: Supplementary file 3 — Description of Additional Supplementary Files [file 41467_2022_28413_MOESM3_ESM.pdf]

Description of Additional Supplementary Files

File name: Supplementary Movie 1

Description: The Supplementary Movie shows the generation and detachment of H_2 bubbles under the increasing overpotentials.
